# Conceptualization of Idle (Laghw) and its relation to medical futility

**Published:** 2016-04-01

**Authors:** Mohsen Rezaei Aderyani, Mohsen Javadi, Saeid Nazari Tavakkoli, Mehrzad Kiani, Mahmood Abbasi

**Affiliations:** 1 Medical Ethics PhD Candidate, Department of Medical Ethics, School of Traditional Medicine, Shahid Beheshti University of Medical Sciences, Tehran, Iran;; 2 Professor, University of Qom, Qom, Iran;; 3 Associate Professor, Department of Medical Ethics, School of Traditional Medicine, Shahid Beheshti University of Medical Sciences, Tehran, Iran.

**Keywords:** *Holy Quran*, *Quran-related dictionary*, *Idle*, *Laghw*, *Medical futility*

## Abstract

A major debate in medical ethics is the request for futile treatment. The topic of medical futility requires discrete assessment in Iran for at least two reasons. First, the common principles and foundations of medical ethics have taken shape in the context of Western culture and secularism. Accordingly, the implementation of the same guidelines and codes of medical ethics as Western societies in Muslim communities does not seem rational. Second, the challenges arising in health service settings are divergent across different countries.

The Quranic concept of idle (laghw) and its derivatives are used in 11 honorable verses of the Holy Quran. Among these verses, the 3rd verse of the blessed Al-Muminūn Surah was selected for its closer connection to the concept under examination. The selected verse was researched in the context of all dictionaries presented in Noor Jami` al-Tafasir 2 (The Noor Collection of Interpretations 2) software.

"Idle" is known as any insignificant speech, act, or thing that is not beneficial; an action from which no benefit is gained; any falsehood (that is not stable or realized); an entertaining act; any foul, futile talk and action unworthy of attention; loss of hope; and something that is not derived from method and thought. The word has also been used to refer to anything insignificant. The notes and derived interpretations were placed in the following categories: A) Having no significant benefit (When medical care does not benefit the patient (his body and/or soul and his life in this world and/or the Hereafter), it is wrong to proceed with that medical modality; B) Falsehood (Actions that fail to provide, maintain, and improve health are clearly futile); C) Unworthy of attention (An action that neither improves health nor threatens it is wrong and impermissible).

## Introduction

Medical ethics can be interpreted as the establishment of the do's and don’ts in medicine. Medical ethics is an interdisciplinary field of knowledge that is associated with various domains of knowledge. The main scientific domain that deals with medical ethics is philosophy. Hence, the foundations of the philosophical school adopted play a critical role in directing recommendations, guidelines, and codes of medical ethics. However, given the profound influence of secularism in general Western culture and in the academic milieu, the foundations of medical ethics in the Western world have been developed based on secular ideology worldview. For example, medical ethics is said to have been secular since the enlightenment of American and European societies (from the 18th century onwards). It is emphasized that this medical ethics does not refer to God or the revelatory tradition and is just focused on the needs and offerings of rational discourse (1). In their "Principles of Biomedical Ethics", Beauchamp and Childress (2) refer to Western philosophical ideas as the basis of ethical analysis. Although this four-principle approach has sought to offer general, comprehensive principles and rules for medical ethics in the form of common morality, the religious and cultural contexts of Western countries and the aforementioned philosophical foundations have inevitably had their influence on it. Based on this framework, principles, rules, guidelines, and codes have been devised; and in specific cases (such as abortion, end-of-life care, euthanasia, and medical futility), results consistent with that particular cultural context have been achieved. 

The advances made in medical technology have provided the opportunity for life-prolonging interventions over the last 50 years. The prolonged life of critically ill patients often depends on devices during the final stages of life, and they do not have a normal connection to the world around them. The increased possibility of interventions has raised new ethical questions (3). In the 1980s, a new term was coined for inappropriate interventions: medical futility (4).

Medical futility is a term used to describe medical interventions that are expected to result in little or no benefit to a patient (5). A major debate in medical ethics is the topic of requesting futile medical care. This issue can threaten the physician-patient relationship (6). Dissipation of medical resources, elimination of or reduction in the opportunity for other patients in need of medical services, erosion of trust in the medical team, and the emergence of legal complexities for the medical team are only a few examples. Although the requests for medical futility compose only a small part of the health system in its totality, they can cause severe psychological and ethical tension for the patient, their family, and the medical team (7). In rare cases, the problem might become so gravely complicated that the matter is taken to the legal authorities by the patient or the hospital (8). 

If we are to analogize the health setting's confrontation with pathogenic factors to a battleground (9), we should recognize that success and failure in this field are not always limited to one front. In this combat zone, there are times when the health domain triumphs and the disease is defeated by the efforts of health service providers and conversely, there are occasions when the tables turn and the disease triumphs. The question, therefore, arises as to how far and to what extent the health setting should mobilize its forces against the disease and pathogenic factors and under which circumstances the health setting should surrender to the disease.

This challenge also rears its head in another guise: the allocation of health resources. For example, the ICU bed and the ventilator are aiding a patient whose imminent death is expected while concurrently, there is a patient in the hospital with a disease amenable to treatment (e.g. Guillain-Barré, a disease that paralyzes the muscles, including the respiratory muscles, for a while, but is reversible and curable) and there is no possibility of setting up another bed and another device. The ventilator is connected to a patient who will die within a few days while another patient is in dire need of the same device to regain his health.

Unrealistic expectations such as "technology is a cure-all" have led to extraordinary demands on the part of patients or substitutes (surrogate decision-makers) to the effect that "anything possible will be done", which can create conflict and disagreements between the health care team and the patient or the substitute relatives (4).

The idea of requesting medical futility is far from being a novel concept. Indeed, Hippocrates advises physicians: "In refusing the treatment of those who have been overcome by their diseases, realize that treatment is powerless." (10).

The important point is that, for at least two reasons, medical futility requires a separate examination in Iran. First, the common principles and foundations of medical ethics have been developed in the context of Western culture (11) and secularism, with secularist values and principles having inescapably permeated all its aspects. There are fundamental differences between secular ontology, anthropology, and epistemology and what is found in a religion-oriented ethical school (12, 13). Based on these differences, planning, goal-setting, and determining ways of achieving objectives are dissimilar in these two worldviews. As the majority of the Iranian population is Muslim and Islam is the country’s official religion (14), the implementation of some doctrines and codes of medical ethics pertaining to Western societies in Muslim communities does not appear to be rational. Muslim scholars (in particular Iranian intellectuals) need to develop a set of ethical principles and values for the Muslim community by careful examination of traditional resources (the revelation and the tradition of the infallibles) and intellectual resources. In accordance with these fundamental values and principles, Muslim medical ethics experts should then develop guidelines and codes of medical ethics for application within a Muslim community. Second, challenges in a health service setting (health and care) vary across different countries. In Iran, major developments have occurred in health services since the Islamic Revolution. The development of human resources and facilities in health services has completely transformed conditions in this sector compared to how it was years ago. Though before the Islamic Revolution, Iran was a country dependent for providing its basic health services, it has now, according to reports from the Ministry of Health and the World Health Organization (WHO), reached acceptable levels with regard to some major indicators of health services and has garnered the praise of the WHO's experts and inspectors. Challenges emerge when instructions, policies, and guidelines have not been developed concurrently with the development of these health services. In other words, the vast majority of efforts have been focused on developing the quantity of services and, apparently, the quality of services has not been addressed in proportion to this quantitative development.

To develop issues of medical ethics in the health system, either the same ideas and principles and codes of Western societies should be translated and instructed in Iran, or, if it is established that the basic foundations and values and, consequently, the codes of Western medical ethics are not consistent with Iranian culture, customs, and religion and are incompatible with the principles and values of the Iranian society, then attempts should be made to prepare and develop codes of medical ethics based on principles and values appropriate to the Iranian society.

As was mentioned above, given that the majority of the Iranian population is Muslim and Islam is the country’s official religion and it seems that Islamic thought and its derived principles may be different from secular thought, this study was planned to investigate the concept of "futility" in the Holy Quran and Quran-related dictionaries with a view to determining the similarities and dissimilarities between these two doctrines.

## Materials and Methods

This paper is a part of a thesis entitled “Medical Futility: a Comparative Study from the Perspective of Secular and Religious Ethics”. Given the subject of "futility" and its conceptual congruence with the Quranic concept of "idle" (laghw), the online software "Pars Quran" (at http://www.parsquran.com/was) and Noor Jami` al-Tafasir 2 software were drawn upon for this word. The root of the word "laghw" and its derivatives are used in 11 honorable verses of the blessed Surahs of the Holy Quran, namely Al-Baqarah: 225; Al-Ma’idah: 89; Maryam: 62; Al-Mu’minun: 3; Al-Furqan: 72; Al-Qasas: 55; Fussilat: 26; At-Tur: 23; Al-Waqi`ah: 25; An-Naba’: 35; and Al-Ghashiyah: 11. Of these honorable verses, the 3rd honorable verse of the blessed Surah of Al-Muminūn was selected for its closer connection to the concept under examination. There is a large congruence between this honorable verse and the subject of "futility". The search results in these software programs yielded no additional honorable verses using the derivatives of the word. The selected honorable verse was searched in all the dictionaries in the Noor Jami` al-Tafasir 2 software (Al-Tahqiq, Qamus al-Quran, Al-Ayn, Lisan al-Arab, Majma’ al-Bahrain, and al-Mufradat). Translations and descriptions provided in these dictionaries with regard to the subject of "futility" were selected from the texts. Arabic texts were translated. Wherever there were doubts about the translation of a word or phrase, the original text and its translation were presented to an Arabic language and literature professor and the translations were readjusted. 

## Results

God Almighty, in honorable verses 1-11 of the blessed Surah of Al-Muminūn, reveals the attributes of believers. According to the 3rd honorable verse of the blessed Al-Muminūn Surah, the second attribute of these meritorious servants is that they avoid being "idle": "*Prosperous are the believers, who in their prayers are humble, and from idle talk turn away*". ^1^

The thematic philology of the word "idle" in the dictionaries shows that it has several meanings. In this study, its closest meaning to the concept of medical futility was chosen as an example. 

According to the blessed Surah of Al-Tahqiq, the word "idle" (laghw) denotes any insignificant speech or act that is of no benefit. It thereafter extends the examples of the word "idle" to more than the word and to external actions and matters. One of the instances that it subsequently provides for the word "idle" is an action from which no benefit ensues and goes on to cite any falsehood or any entertaining act as examples of "idle". In addition, it claims "idle" to be futile and against that which is right and stable (15).

Here, the author refers to honorable verse 3 of the blessed Surah of Al-Muminūn ("*and from idle talk turn away*") as an example. He interprets the honorable verse as stating that, "a believer always proceeds with his life under the religion of the Almighty God and acts according to Divine directions and commands and prohibitions. He is an obedient servant to his Lord who does not neglect his duties of servitude even for a moment. He believes that the Almighty God perceives his position and hears his words and the truth is nothing but that his good and bad deeds all get back at himself in this world and the Hereafter. So how is it possible for him to do anything futile that stops him from devoting himself to the Almighty God, that prevents him from fulfilling his duties of servitude and that acts as a barrier between him and his Lord?"(15). 

By referring to honorable verses 52-55 of the blessed Surah of Al-Qasas (*"Those to whom We gave the Book before this believe in it ... When they hear idle talk, they turn away from it and say, 'We have our deeds, and you your deeds. Peace be upon your We desire not the ignorant.*"), the author does not limit the avoidance of "idle" to the believers of Islam and extends it to all followers of the book. Based on honorable verses 71 and 72 of the blessed Al-Furqan Surah (*"And whosoever repents, and does righteousness, he truly turns to God in repentance. And those who bear not false witness and, when they pass by idle talk, pass by with dignity.*"), he claims the importance of avoiding "idle" as an important attribute for Ibad al-Rahman (the servants of the All-Merciful), coming in the order after repentance and negating false testimony (15). 

By referring to honorable verse 25 of the blessed Surah of Al-Waqi`ah ("*Therein they shall hear no idle talk, no cause of sin*"), honorable verse 35 of the blessed Surah of An-Naba’ (*"Therein they shall hear no idle talk, no cry of lies*"), and honorable verses 10 and 11 of the blessed Surah of Al-Ghashiyah ("*in a sublime Garden, hearing there no babble*"), the author claims that the inhabitants of Paradise do not hear an idle word and considers this state one of the attributes of the inhabitants of Paradise (15).

Ghamoos al-Quran defines "idle" as useless speech and states that "laghiya" is a disagreeable speech and "loghat" (language) was given its name because it is useless for non-speakers. Then, by referring to honorable verse 89 of the blessed Surah of Al-Ma’idah (*"God will not take you to task for a slip in your oaths; but He will take you to task for such bonds as you have made by oaths*"), he claims that idle oath is an oath without an intention such as the expressions "Wallah" (I swear to God) and "Billah" (with God or through God), which are used habitually in speaking, and to take an oath is to strengthen it by an intention and to swear intentionally and consciously and the verse means that God will not punish you for what is unintentional in your oaths, but He will punish you for those oaths you have strengthened by your intention (16).

By referring to the 3rd honorable verse of the blessed Surah of Al-Muminūn, the author recognizes the meaning of "idle" as "apparently every futile word and action". He also refers to honorable verses 10 and 11 of the blessed Surah of Al-Ghashiyah: "*in an elevated garden, wherein they will hear no unsuitable speech*". Therefore, taking an unintentional oath not intertwined with the person's soul and not expressed with serious intent and out of faith is void and is considered idle, and any disagreeable, useless word and action will be also idle (16).

The Al-Ayn dictionary also takes the word "idle" to mean falsehood and, by making reference to honorable verse 26 of the blessed Surah of Fussilat ("*The unbelievers say, 'Do not give ear to this Koran, and talk idly about it; haply you will overcome'*."), it recounts the words of the enemies of Islam and defines "idle" in this verse as the raising of their voices so as to confuse the Muslims (17).

According to Lisan al-Arab, "idle" is interpreted as a useless, futile word or any other thing not worth paying attention to and not beneficial, or any speech that is irrelevant and off-topic. "Idle" has also been taken to mean deviation from the right path and the state of getting disappointed (18).

In trying to define the word "idle", Majma’ al-Bahrain refers to honorable verse 57 of the blessed Surah of Al-Furqan and takes "idle" to mean the same concept as falsehood. Subsequently, by referring to honorable verse 26 of the blessed Surah of Fussilat, in which non-believers advise one another, the author states that, in this verse, "*and talk idly about it*", the word "idle" can be defined as obsolete speech in which there is no benefit (19). 

Al-Mufradat fi Gharib-e Al-quran writes: "It is an insignificant speech and anything not derived from method and thought … and any disagreeable word is called idle. And it is used for anything insignificant" (20).

  Quran: 23: 1-3َ‏ 

## Discussion

Considering the findings from dictionaries, it seems necessary to examine whether or not the interpretations provided for the word "idle" in the dictionaries are related to medical ethics, in particular the issue of "futility". For this purpose, the present findings will be compared to cases that are their equivalent in a health service context, and their similarities and congruities will be discussed.

Based on Tafsir al-Jalalayn in the commentary of the verse “and from idle turn away”, idle includes speech and other things (21). It is also stated in the Tafsir of Qurtubi that idle includes any void and unseemly word or act, like ghena music, useless actions, erotic conversations, and other sins and obscenities (22). According to Tabari, in Arabic language, any void act or speech is known as idle (23). Ibn Kathir has mentioned in his Quran’s Tafsir that useless acts or words are called idle (24). The fact that the concept of idle is not restricted to speech and, as a general concept, includes acts too is so obvious that it is mentioned in non-interpretational works, too. For example, in his book, Shu’ab al-Iman, Ahmad ibn Husain Bayhaqi (d. 456) explains different instances of idle including in them wasting time in useless matters, revealing people’s secrets, abuse, performance of magic, and composing hyperbolic poetry (25). This generality of meaning is so clear that the Author of Mashahir ‘Ulama al-Amsar regards acts such as unlawful trade and perjury as instances of idle (26).

**A) Having no significant benefit**

In Al-Tahqiq, Ghamoos al-Quran, Lisan al-Arab, and Majma’ al-Bahrain dictionaries (15, 16, 18, 19), the word "idle" is said to be any work that has no benefit or significant benefit. One of the four common ethical tenets of medicine in the world is the principle of beneficence, and in clinical decision-making, if a patient’s benefit is in conflict with other affairs, his benefit takes priority. Thus, medical futility is wrong as it is not beneficial to the patient. The mentioned principle is not purely in contrast with religious doctrines; nevertheless, in religious morality, cases of benefit and harm and their domain are broader than what is perceived in a merely materialistic view. From the perspective of religious morality, man is an eternal creature who will not perish with death; rather, he sheds his skin (27). The comprehensive perspective of religion on the dimensions of human life necessitates that the determination of the individual’s benefits not be grounded only on bodily benefits and transient worldly life. These doctrines take other dimensions of human existence (such as the soul or Divine breath) and man’s eternal life in the hereafter as the criteria for determining benefit and harm. If the treatment intended does not have a significant benefit for any of the dimensions of human existence, the provision of that treatment is wrong and impermissible in Islamic morality and it is obligatory that it be avoided. It is now clear that the examples provided in the mentioned dictionaries for the word "idle" not only have no significant benefit for man, but are also harmful and damaging and should, therefore, be avoided. 

**B) Falsehood**

Al-Tahqiq examines the philology of the word "idle" and takes it to be falsehood and against that which is right, realized, and stable (15). In health service settings, if estimations indicate the unjustifiability and impossibility of any result being achieved through the intended treatment, the treatment is considered futile, false, and idle.

Another point is the interpretation supplied by Al-Mufradat fi Gharib al-Quran. The author defines the word "idle" as anything that is not derived from method and thought (20). Medical science is an empirical science, and evidence-based medicine has become a valuable part of medicine. "Futility" has been defined as a treatment not falling within the range of medical standards and, therefore, being deemed medical futility (28). Accepted evidence-based treatments have been derived from a thought-based procedure; consequently, offering treatments outside the range of medical standards is considered "futile" according to this definition. 

The writer of Lisān al-Arab uses another signification for the word "idle" and says, "An irrelevant and off-topic speech … Idle has also been taken to mean deviation from the right path." (18). This signification can be taken as physiologic futility, which is a type of futility. (9, 11, 29)

**C) Unworthy of attention**

According to Lisān al-Arab, the word "idle" denotes pointless, useless speech and anything else that is not worth noting and has no benefits in it (18). This signification can be taken as evaluative futility, which is considered a type of futility in the available literature on the subject (9, 30).

## Conclusion

**A) Having no significant benefit**

One of the four tenets of medical ethics presented by Beauchamp and Childress, which are accepted and well-known in many countries, is the principle of beneficence. In addition, based on ethical policies such as the Geneva Declaration, in clinical decisions, the patient’s benefit is always a priority. In health service settings, any action that is not beneficial to the patient based on narrative or rational and empirical evidence is unacceptable and the patient and service providers need to cease pursuing it. Accordingly, since medical futility is not beneficial to the patient, it is inconsistent with the two principles of beneficence and non-maleficence and is, as such, wrong. The following presents instances of measures which are not planned based on the prioritization of the patient’s benefit: Simultaneously treating multiple differential diagnoses for the patient’s clinical status; Administrating antibiotics for common colds, which are most likely viral; Administrating injection drugs when non-injection drugs can be used instead; Prescribing a test without checking the patient's history and performing a thorough physical examination; Performing endoscopy without appropriate clinical criteria; and Hospitalizing a patient who can be treated and followed up as an outpatient.

In health settings, pursuing therapeutic-diagnostic measures without documented medical reasons (particularly with the sole motivation of financial gain or fame) is wrong. Examples provided are instances of taking measures without a reason, which are unacceptable and impermissible according to both secular ethics and particularly from the perspective of religious ethics. A faithful physician with strong religious beliefs respects the command and prohibition dictated by the holy law (command and prohibition of the intellect, The Book, and the Tradition) based on observing the right of God, and respects the patients’ rights based on observance and accountability to the right of people, and envisions such a great right of self and honor that he avoids engaging in such activities and performing therapeutic-diagnostic measures without documented medical reasons (particularly for the sole motivation of financial gain).

**B) Falsehood**

The administration of antibiotics for viral diseases (such as most common colds) is ineffective in bringing health and is, therefore, futile and should be avoided from the perspective of Islamic ethics.

Consequently, a treatment not included in medical standard, not derived from method and thought, not accepted through evidence-based medicine, and/or not having any physiologic effect on the improvement of man’s health is false, non-relevant, and, therefore, futile.

When a patient visits a physician, one of the questions that need to be answered is whether the patient requires hospitalization or if he will recover through outpatient treatments without any serious complications. One of the cases that may be witnessed in health centers is the patients’ hospitalization without indication. The total costs of outpatient treatments are often less than the costs of hospitalization. The general culture in our country (as is the case in many Asian countries) is family-oriented. The lack of human resources (particularly nurses) in addition to the family-oriented culture necessitates the presence of a relative of the hospitalized patient in the hospital as a companion. The costs of unnecessary hospitalization thus impose a heavy burden on the patient’s relatives, as well. When the patient does not benefit from hospitalization, his hospitalization is morally impermissible and wrong regardless of the motivations behind it. 

**C) Unworthy of attention**

Whenever according to medical assessments, the considered treatment results in outcomes such as prolonged life for hours, days, months, and even years, but according to those estimates, the result is of no substantial value; this is evaluative futility. Evaluative futility is one of the challenges of clinical cases. The patient and his family may regard the medical measure as a worthwhile action, whereas the medical practitioner, albeit aware of the possibility of increased longevity of the patient's life, may deem this longevity worthless. In this case, one of the major challenges is whose values and generally what types of values should constitute the criteria for action.

In religious teachings, patience is a moral virtue. The Holy Quran commands the Dear Prophet of Islam (PBUH) to give "*good tidings to the patient*" (Al-Baqarah, 155). "*Allah loves the steadfast*." (Ali Imran, 146). "*Indeed, Allah is with the patient*." (Al-Baqarah, 153 & 249; Al-Ma’idah, 46 & 66). "Patience during calamity", which is one of the types of patience, is acceptable and worthy, and disease is considered a test and a calamity. When in determining individual interests, the interests of the body and the spirit as well as the worldly life and the eternal life of man are taken into consideration, it can be perceived that requests for euthanasia take away the opportunity of practicing patience during illness from the patient/family. Therefore, such measures are not beneficial to the patient/family and are morally wrong. 

From what was said, it can be concluded that there are several significations for the word "laghw" (idle) in the examined dictionaries. Examples of these significations include any insignificant speech or thing, any action from which no benefit ensues, falsehood, entertaining act, anything wrong and against the right that is not stable or realized, useless speech, any disagreeable and useless speech and action, any speech that is irrelevant and off-topic, deviation from the right path, getting disappointed, and also anything not derived from method and thought.

It can thus be concluded that despite superficial similarities, the religious thought (and in specific, Islamic thought) is deeply different from secularism. Believing in and adhering to the three basic principles of unity, prophethood, and resurrection is in apparent contradiction to the principles and values of secularism. It is necessary for Muslim and Shia scientists to collect their own set of moral principles and values for implementation in Muslim communities after careful examination of the traditional resources of their culture (Revelation and Tradition of the Infallibles) as well as their intellectual resources. They should then formulate medical guidelines and ethical charters for Muslim communities based on these more harmonious principles and fundamental values.
